# Evaluation of acute plant toxicity, antioxidant activity, molecular docking and bioactive compounds of lemongrass oil isolated from Omani cultivar

**DOI:** 10.1016/j.toxrep.2024.101888

**Published:** 2025-01-05

**Authors:** Haneen Al Weshahi, Mohammad Sohail Akhtar, Salem Said Al Tobi, Amzad Hossain, Shah Alam Khan, Alia Bushra Akhtar, Sadri Abdullah Said

**Affiliations:** a Shinas extended health Centre MOH, Oman; b School of Pharmacy, College of Health Sciences, University of Nizwa, Oman; c Department of Pharmaceutical Chemistry, College of Pharmacy, National University, Oman; d Department of Translational and Clinical Research, School of Chemical and Life Sciences, Jamia Hamdard, New Delhi, India

**Keywords:** Lemongrass, Citral, Volatile oil, GC-MS, Cytotoxic: Antioxidant

## Abstract

Lemongrass (Poaceae) is one of the aromatic plants with strong odors. Traditionally, lemon grass oil has been used for the treatment of many diseases such as gastrointestinal cramps, high blood pressure, high body temperatures, and fatigue, and is also considered an antibacterial and anti-diarrheal agent. Therefore, this study aims to investigate volatile active constituents and a few important biological activities of the volatile oil of lemongrass (Cymbopogon citratus) grown in Oman. To support the results of experimental studies, and to find out the main active constituents responsible for exhibiting biological activities molecular docking studies have also been performed. A sufficient amount of essential oil was obtained using steam distillation from fresh leaves of lemongrass. Volatile constituents were identified with the GC-MS analysis. Lemon grass oil exhibited a very good *in vitro* antioxidant activity (65.08–90.48 % inhibition of DPPH) with increasing concentration (31.25–1000 µg/mL) of oil. Isolated oil also exhibited good cytotoxic activity against the brine shrimps (100 % mortality at 1000 mcg/mL). Furthermore, molecular docking studies confirmed that beta citral is the monoterpene compound responsible for antioxidant and cytotoxic activity.

## Highlights

• Successfully isolated a substantial amount of volatile oil from fresh lemongrass.
• Identified active compounds using GC-MS analysis.
• Demonstrated strong antioxidant activity of the lemongrass oil.
• The volatile oil exhibited notable cytotoxic activity against brine shrimp larvae.
• Docking studies confirmed that beta citral is a key monoterpene responsible for antioxidant and cytotoxic activities.

## Graphical Abstract

**Figure:**
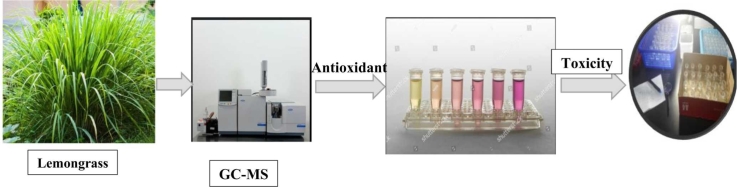

## 1 Introduction

*Cymbopogon citratus* (*C. citratus* Family: Poaceae) is commonly known as lemongrass. This aromatic plant belongs to a genus that includes approximately 55 species. As a perennial grass, lemongrass thrives predominantly in tropical and subtropical regions worldwide. Research indicates that its origins trace back to Asia, particularly in areas such as Indochina, Indonesia, and Malaysia, as well as parts of Africa and the Americas [1]. Today, it is cultivated in various tropical and subtropical regions, including Asia, South America, Central America, and certain tropical countries in Africa [2]. The distinctive lemon scent of lemongrass is attributed to its high citral content, which is a key component contributing to its aromatic properties. Beyond its pleasant fragrance, lemongrass exhibits notable antimicrobial activity and offers a range of health benefits, including treatment for digestive disorders, diabetes, nervous system issues, and even cancer [3].

*C. citratus* is a perennial herb that typically grows to a height of about one meter, characterized by its tough, leafy stems. The leaves are long, shiny, and green, tapering to a pointed tip. During its growth stage, lemongrass produces flowers, although these are not the primary focus of its cultivation. The bulbous stems feature terete (cylindrical) and glabrous (smooth) sheath leaves that are linearly vented, with a narrow base leading to an acute tip. The leaves can reach approximately 100 cm in length and 2 cm in width, contributing to the plant's distinctive appearance [4].

Lemongrass tea, made from its leaves, is popular in regions such as South America, Asia, and West Africa. This infusion is valued for its antibacterial, anti-fever, anti-dyspeptic, carminative, and anti-inflammatory properties [5].

Lemongrass oil is renowned for its pleasant and refreshing aroma, along with its antifungal and antibacterial properties. This essential oil is widely imported by the United States and European countries [5]. The oil contains a variety of chemical compounds, including hydrocarbon terpenes, alcohols, ketones, esters, and aldehydes. Notably, it features bioactive constituents such as myrcene, which is recognized for its antibacterial and pain-relieving effects. The primary terpenoid in lemongrass oil is citral, a mixture of two stereoisomeric aldehydes [6]. This oil serves multiple purposes; it is often used as incense and has numerous medicinal benefits, including antibacterial and antipyretic actions. Various studies have reported that lemongrass oil can help improve digestion, alleviate nausea, and relieve ailments such as headaches and muscle cramps [7]. In terms of medicinal applications, it is commonly used to treat gastrointestinal cramps, high blood pressure, elevated body temperature, and fatigue, and is also recognized for its antibacterial and anti-diarrheal properties. Industrially, lemongrass oil is utilized in the manufacture of soap and beauty products, as well as in the production of natural citral [8]. In one analysis, lemongrass oil was found to contain 28 different compounds, with neral and geranial being the major constituents, together contributing up to 72 % of the total composition. The concentration of geranyl acetate (GA) is approximately 3.5 %. Other notable constituents include limonene, β-myrcene, linalool, β-caryophyllene, methyl eugenol, gramecene-D, caryophyllene oxide, and δ-cadinene [9].

The essential oil of lemongrass exhibits a wide variety of biological activities, including insecticidal, antibacterial, antifungal, antiviral, cytotoxic, and antioxidant effects [10]. A study conducted in India in 2014 investigated the antibacterial activity of lemongrass extracts. The results revealed that all solvent extracts, regardless of polarity, effectively inhibited *Pseudomonas aeruginosa*, *Bacillus subtilis*, and *Proteus vulgaris* to a significant extent. However, *Staphylococcus aureus*, *Nocardia* sp., *Enterobacter aerogenes*, and *Serratia* sp. were inhibited to a lesser extent by the extracts. The antibacterial activity of *C. citratus* leaf extracts was found to be effective against both Gram-positive and Gram-negative bacteria [11], [12], [13], [14], [15], [16], [17], [18]. While lemongrass is known globally for its benefits, there may be a lack of comprehensive studies focusing specifically on the varieties cultivated in Oman. Understanding the unique properties and potential of locally grown lemongrass is crucial. Despite its known medicinal benefits, there may be limited awareness or use of lemongrass and its essential oil in local healthcare practices or traditional medicine in Oman. Oman may not fully leverage the economic benefits of cultivating and exporting lemongrass oil and its byproducts. Identifying and promoting its value could enhance local economies and create new markets. By addressing these problems, your study can contribute to a better understanding of lemongrass in Oman, promote its benefits, and potentially enhance local agricultural practices and economic opportunities. Therefore, this study aims to investigate the volatile active constituents and key biological activities of the essential oil extracted from lemongrass cultivated in Oman. Additionally, the research seeks to identify the main active compounds responsible for these biological activities. To support these findings, molecular docking studies will also be conducted to elucidate the interactions between these compounds and potential biological targets.

## 2 Materials and methods

### 2.1 Sample collection

Fresh leaves of lemongrass were collected from the Shinas region (The latitude and longitude of Shinas, Oman is: 24° 43' 48" N / 56° 27' 46" E) and thoroughly washed under running water to remove any contaminants. The plant was then identified by a taxonomist Assoc. Prof. Dr. Syed Abdullah Gilani, Department of Biology and Chemistry, University of Nizwa, Oman. The voucher specimen (0198) was deposited in the Natural Product Research Lab, School of Pharmacy, University of Nizwa, Oman

### 2.2 Isolation of volatile oil

Steam distillation was employed to isolate the essential volatile oil from the fresh leaves of lemongrass (*Cymbopogon citratus*). In this process, steam is passed through the plant material, causing the essential oils to evaporate. The steam and oil vapors are then condensed back into liquid form. The essential oil is separated from the water, resulting in a pure extraction of the volatile compounds present in the lemongrass leaves. The isolated volatile oil was analyzed using a Gas Chromatography-Mass Spectrometry (GC-MS) instrument. Each compound present in the oil was identified by comparing its mass spectra with standard spectra in a computer database.

### 2.3 GC-MS instrument and analysis program

The isolated essential oil was analyzed using a Gas Chromatograph (Bruker, Model 7890 A) coupled with a Mass Spectrometer (MSD, Agilent, Model 5975), fitted with an Agilent HP-5MS IU capillary column (30 m × 0.25 mm ID × 0.25 µm film thickness). Helium gas (purity 99.999 %) was employed as the carrier gas at a constant flow rate of 1 mL per minute. The temperature settings were as follows: the injector temperature was set to 240°C, the transfer line to 237°C, and the ion source to 230°C. The oven temperature program began at 100°C (held for 3 minutes) and increased to 240°C at a rate of 10 °C/min, with a hold of 3 minutes at the final temperature. A sample volume of 0.1 µL was injected into the GC injector using a splitless mode. Data were collected in full scan mode with a mass range of 50–550 amu. The collected data were processed using Mass Hunter software (version 10). Identification and characterization of each compound in the essential oil were based on retention times and comparisons with in-built mass libraries (NIST).

### 2.4 Free radical scavenging assay

The antioxidant activity of lemongrass oil was assessed using the Brand Williams method [19] with modifications involving the DPPH radical scavenging assay. Six concentrations of the isolated oil were prepared in hexane: 31.25, 62.5, 125, 250, 500, and 1000 µg/mL. For each concentration, 3 mL of the oil solution was mixed with 1 mL of a 0.3 mM DPPH hexane solution. The reaction mixture was vigorously shaken and then incubated in a dark place at room temperature for 30 minutes. The absorbance of each sample was measured at 517 nm using UV-Visible spectrophotometer. A control sample was prepared without the addition of oil. The radical scavenging activity of the tested concentrations was calculated using the following formula:

$$\%\mathrm{RSA}=\;\frac{A_{o}-\;A_{s}}{A_{o}}\;\times 100$$

Where A_o_ is the absorbance of the control and A_s_ is the absorbance of the test sample [19].

### 2.5 Cytotoxic activity

#### 2.5.1 Cytotoxicity assessment

Cytotoxicity was evaluated using brine shrimp (*Artemia salina Leach*) larvae [20], [21], [22], [23], [24], [25].

#### 2.5.2 Hatching of shrimp larvae

Approximately 50 mg of brine shrimp eggs were spread across prepared seawater in a small polyethylene tank, which was divided into two compartments. The seawater was prepared by dissolving 38 g of sea salt. One compartment remained dark, while the other was illuminated to attract the shrimp larvae from the dark chamber after they hatched, which occurred within 24 hours.

##### 2.5.2.1 Brine shrimp lethality test

A 10 mg/mL stock solution of volatile oil in dichloromethane was diluted to prepare test concentrations of 31.25, 62.5, 125, 250, 500, and 1000 µg/mL. The dichloromethane solvent was then evaporated. Around 3 mL of artificial seawater was added to each vial, followed by the addition of 10 shrimp larvae per vial. The solution was then diluted to a final volume of 5 mL using artificial seawater. The vials were maintained at room temperature and illuminated for 24 hours. The experiment was conducted in triplicate. After 24 hours, the number of live larvae was counted, and the percent mortality of the larvae was calculated.

##### 2.5.2.2 Data analysis

The LC50 values for each sample were determined by probit analysis using the "EPA Probit Analysis Version 1.5" software [26].

### 2.6 Molecular docking studies

**Fig. 1:**
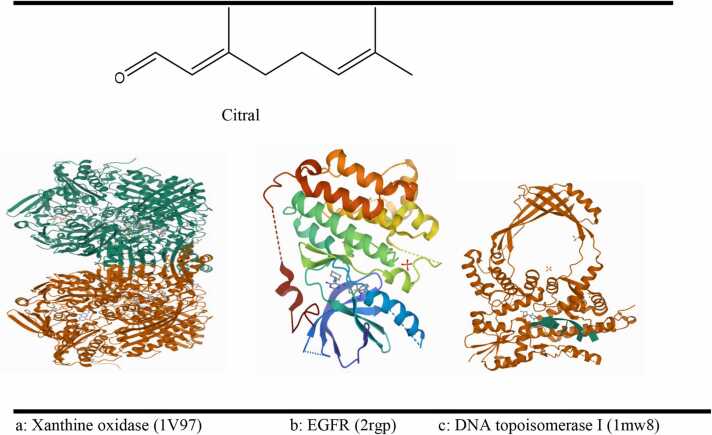
2D and 3D structure of citral, Xanthine oxidase, EGFR and DNA topoisomerase I.

**Fig. 2:**
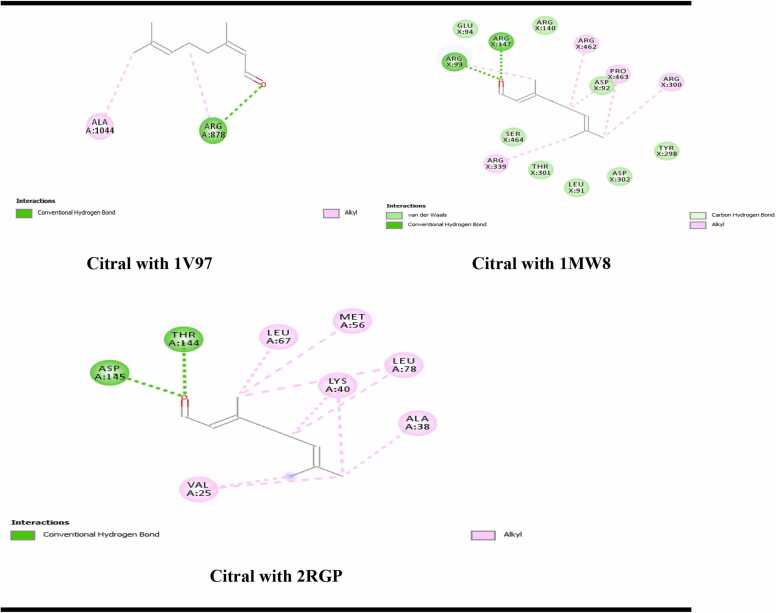
2D interactions of citral with 1V97, 2RGP and 1MW8.

Molecular docking was conducted using PyRx with AutoDock Vina, employing a semi-flexible docking system. The chemical structure of beta citral was downloaded from the PubChem database in SDF format (Fig. 1). The 3D crystal structures of three proteins were retrieved from the RCSB Protein Data Bank in PDB format: (a) Xanthine oxidase (1V97), (b) EGFR (2RGP), and (c) DNA topoisomerase I (1MW8) (Fig. 2). Water molecules and ligands were removed using UCSF Chimera, and the structures were saved in PDB format. The proteins were prepared for docking using AutoDock Vina tools in PyRx and were saved in PDBQT format. Each structure underwent energy minimization and was subsequently saved in PDBQT format. The docking results, including protein-ligand interactions, were analyzed using Biovia Discovery Studio, and the binding energy was reported in kcal/mol [27].

## 3 Results and discussion

Lemongrass has a variety of traditional and contemporary medicinal uses, making it a valuable herb in both culinary and therapeutic applications. Lemongrass is often used to alleviate digestive issues, such as bloating, constipation, and indigestion. It is known for its carminative properties, which help in reducing gas and promoting digestion. The essential oil of lemongrass exhibits antibacterial and antifungal properties, making it useful in treating infections and preventing microbial growth.

### 3.1 Isolation of volatile oil

One kilogram of fresh lemongrass was subjected to steam distillation for the isolation of volatile oil. The process yielded 1 mL of volatile oil, resulting in a percentage yield of 0.01 %.

### 3.2 GC/MS analysis

The isolated oil was injected into the GC injector using a splitless mode. Data were collected in full scan mode and subsequently processed using Mass Hunter software (version 10). The identification and characterization of each compound in the essential oil were based on retention times and comparisons with in-built mass libraries (NIST). The list of identified compounds, along with their retention times and percentage composition in the essential oil, is presented in Table 1. The analysis revealed the presence of 17 volatile compounds in the isolated oil, with four major compounds classified as monoterpenes. The principal compounds identified in the oil include geraniol (6.37 %), β-myrcene (12.6 %), β-citral (31.03 %), and citral (35.8 %). Among these, citral was found to be the most abundant, constituting 66.83 % of the total oil (Table 1). These compounds have applications in medicinal**,** aromatherapeutic, and cosmetic industries, helping with a range of inflammatory**,** digestive**,** and infectious ailments, in addition to their more common use in flavoring and fragrance[4], [7].

**Table 1:** GC-MS analysis of isolated lemongrass oil.

**No**	**Compound name**	**Rt (min)**	**Area**	**Area %**	**KI (NIST)**	**KI (calculated)**
1	5-Hepten−2-one, 6-methyl-	10.213	11783356	1.69	985	988
2	β-Myrcene	10.359	87924654	12.6	979	992
3	trans- β-Ocimene	11.991	5638717	0.81	1034	1039
4	β-Ocimene	12.351	3129947	0.45	1041	1049
5	Linalool	14.181	8486072	1.22	1100	1101
6	6-Octenal, 7-methyl−3-methylene-	15.766	4421337	0.63	1146	1146
7	Citronellal	16.053	1446102	0.21	1133	1154
8	Isoneral	16.463	14590964	2.09	1151	1166
9	Rose furan oxide	16.845	974179	0.14	1161	1177
10	Carveol	16.973	2000527	0.29	1188	1181
11	3,7-Dimethyl−3,6-octadienal	17.093	19525136	2.8	1184	1184
12	Citronellol	18.707	6235010	0.89	1208	1232
13	β -Citral	19.174	21724808	31.03	1214	1246
14	Geraniol	19.596	44422747	6.37	1232	1258
15	Citral	20.196	25432708	35.8	1240	1276
16	Geranic acid	22.835	1801515	0.26	1358	1357
17	Geranyl acetate	23.734	10524125	1.51	1360	1386

### 3.3 Antioxidant activity

Antioxidants are vital for protecting against oxidative damage, playing a key role in repairing or removing damaged molecules in all aerobic organisms, including humans. In this study, we examined the free radical scavenging activity of lemongrass volatile oil isolated from a local cultivar grown in Shinas, northern Oman, using the DPPH assay. The isolated volatile oil demonstrated increasing levels of antioxidant efficacy in a dose-dependent manner. Specifically, the lemongrass volatile oil exhibited antioxidant activity, with DPPH inhibition ranging from 65.08 % to 90.48 % at concentrations of 31.25–1000 µg/mL. This antioxidant activity is likely attributed to the presence of various terpenoid compounds [18]. The results of the antioxidant activity, including the percentage of inhibition, are presented in Table 2. The highest antioxidant activity was obtained from 1000 µg/mL and the lowest was in 31.25 µg/mL. The lemongrass oil shows significant promise as an antioxidant agent, with its effectiveness increasing as the concentration rises. This activity is likely driven by the terpenoids in the oil, particularly citral, which is known for its antioxidant and other therapeutic benefits [16]. These results support the potential use of lemongrass oil in healthcare and cosmetic products aimed at reducing oxidative damage and promoting overall skin and cellular health [23].

**Table 2:** Percentage of DPPH inhibition by lemongrass volatile oil.

Concentration (µg/mL)	Inhibition (%)
31.25	65.08 ± 0.09
62.5	84.13 ± 0.15
125	85.71 ± 0.24
250	87.30 ± 0.10
500	88.89 ± 0.19
1000	90.48 ± 0.56

DPPH: 2,2-diphenyl-1-picrylhydrazyl

### 3.4 Cytotoxic activity

The percentage (%) of mortality of brine shrimp larvae exposed to various concentrations of volatile oil obtained from lemongrass is presented in Table 3. The results indicate that lemongrass volatile oil exhibits significant activity against brine shrimp larvae. The oil demonstrates effectiveness starting from a concentration of 125 µg/mL to 1000 µg/mL, with percentage mortality ranging from 10 % to 100 %. Notably, the percentage of mortality increases with the concentration of volatile oil, with maximum mortality reaching 100 % at a concentration of 1000 µg/mL. The brine shrimp lethality assay reveals that lemongrass volatile oil demonstrates a concentration-dependent toxic effect, with 100 % mortality at the highest concentration of 1000 µg/mL. While this supports its potential utility in applications like pest control, it also underscores the need for careful consideration of dosage and safety when exploring its use in human health products [9], [25].

**Table 3:** Mean mortality of brine shrimp larvae when exposed to lemongrass volatile oil (n = 10 larvae per treatment).

**Concentration (µg/mL)**	**Mortality (%)**
31.25	0
62.5	0
125	10
250	80
500	90
1000	100

n = Number of larvae.

#### 3.4.1 LC_50_ value of lemongrass volatile oil against brine shrimps’ larvae

The LC50 value for lemongrass volatile oil, determined from probit analysis results, is presented in Table 4. The LC50 value of lemongrass volatile oil is 194.63 µg/mL, indicating its potency. An LC50 value of less than 200 µg/mL for any plant extract or volatile oil is generally considered to signify high activity. Therefore, the volatile oil of lemongrass, with an LC50 of 194.63 µg/mL, demonstrates significant cytotoxic activity and shows potential for application in cancer treatment. This suggests that lemongrass oil, particularly through its active compounds like citral**,** could hold promise as an anticancer agent. However, further studies, especially in human cell lines and clinical trials, would be necessary to validate its therapeutic potential**,** safety, and efficacy in cancer treatment.

**Table 4:** Probit analysis of mortality (LC_50_) of Lemongrass volatile oil against brine shrimp larvae (n = 10).

**Extract**	**LC**_**50**_
Lemongrass oil	194.632591

**LC**_**50**_**=** Lethal Concentration 50

### 3.5 Molecular docking studies of selected active constituents from lemongrass volatile oil

**Fig. 3:**
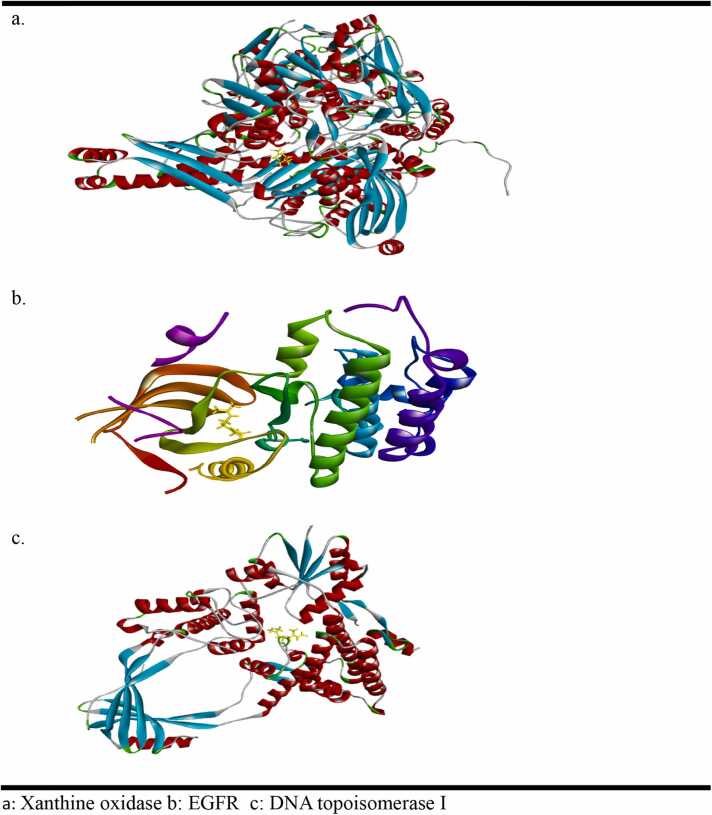
Citral in the binding pocket of (a) Xanthine oxidase (1V97), (b) EGFR (2rgp) and (c) DNA topoisomerase I (1mw8).

The binding energy results presented in Table 5 indicate that beta citral, the major constituent of the isolated oil, binds more strongly to the EGFR with a binding energy of −5.9 kcal/mol compared to DNA topoisomerase I, which has a binding energy of −5.3 kcal/mol. This suggests that beta citral may produce cytotoxicity by inhibiting both EGFR and DNA topoisomerase I. Additionally, it demonstrated strong binding affinity to xanthine oxidase, which correlates well with the in vitro antioxidant results (Figs 1–3).

**Table 5:** Results of ligand and target proteins (PDB: 2rgp, 1mw8 and 1v97) interactions.

**S. No**		**Binding energy (Kcal/mol) of ligand interaction with protein**		
		**1mw8 (DNA topoisomerase I)**	**2rgp (EGFR)**	**1v97 (xanthine oxidase/ dehydrogenase)**
1.	Name of ligand: Beta Citral	−5.3	−5.9	−5.8
2	Organism	*E. coli*	Homo sapiens (human)	Bos taurus
3	Resolution	1.90 Å	2.0 Å	1.94 Å
4	Method used	X ray diffraction	X ray diffraction	X ray diffraction

1mw8 = DNA topoisomerase I 1v97 = Xanthine oxidase

### 3.6 DNA topoisomerase I (1mw8) with citral

**Fig. 4:**
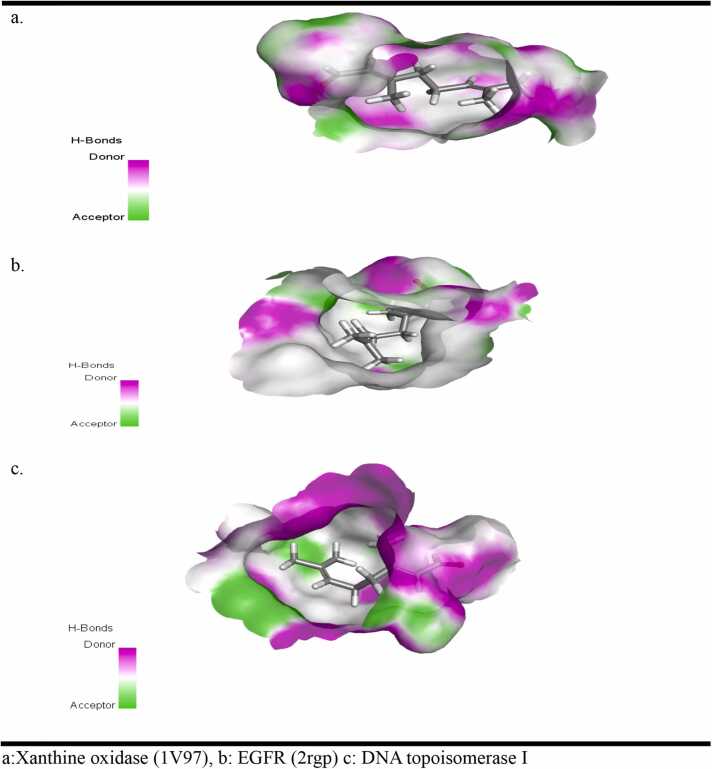
Hydrogen bond surface of proteins (a)Xanthine oxidase (1V97), (b) EGFR (2rgp) and (c) DNA topoisomerase I (1mw8) with citral.

**Fig. 5:**
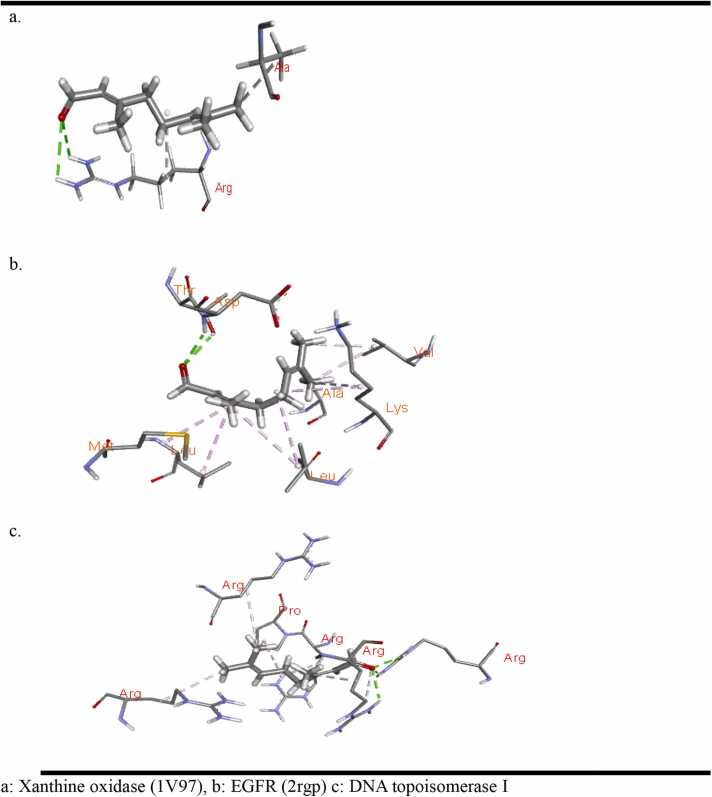
Interaction of amino acid residues of (a) Xanthine oxidase (1V97), (b) EGFR (2rgp) and (c) DNA topoisomerase I (1mw8) with citral.

Citral formed one hydrogen bond through its carbonyl group with the ARG878 residue and an alkyl bond with the ALA1044 residue of xanthine oxidase (Fig. 2). In contrast, when interacting with the EGFR protein (2RGP), citral established two hydrogen bonds with the ASP145 and THR144 residues, along with six alkyl interactions with various amino acid residues (Fig. 2). Although citral formed two hydrogen bonds (with ARG93 and ARG140) and several alkyl and van der Waals interactions with DNA topoisomerase I (1MW8), the binding energy score for this interaction was lower than that for EGFR (Fig. 3). This indicates that citral inhibits EGFR more effectively than topoisomerase I. The hydrogen bond surface, ligand interactions with residues in the binding pocket, and the ligand positioning within the binding pocket of the proteins are illustrated in Fig. 3, Fig. 4, Fig. 5.

## 4 Conclusion

An adequate amount of volatile oil was successfully obtained from fresh leaves of lemongrass. The active constituents were identified using GC-MS analysis. Notably, a strong antioxidant activity was observed, which increased with higher concentrations of the oil. Additionally, significant cytotoxic activity was noted against brine shrimp. Molecular docking studies confirmed that beta citral is the monoterpene compound primarily responsible for both the antioxidant and cytotoxic activities.

## Finding

The authors did not receive any sought of research grant or fund from any place for the completion of this present study.

## CRediT authorship contribution statement

**Haneen Al Weshahi:** Formal analysis, Data curation. **Dr. Mohammad Sohail Akhtar:** Supervision, Project administration. **Salem Said Jaroof Al Touby:** Writing – review & editing, Resources. **Md Amzad Hossain:** Writing – review & editing, Writing – original draft, Project administration. **Shah Alam Kha:** Formal analysis, Data curation. **Alia Bushra Akhtar:** Formal analysis, Data curation. **Sadri Abdullah Said:** Validation, Methodology, Investigation.

## Declaration of Competing Interest

The authors declare the following financial interests/personal relationships which may be considered as potential competing interests. Dr Amzad Hossain reports was provided by NA. Dr Salem Shadeed reports administrative support, equipment, drugs, or supplies, statistical analysis, and writing assistance were provided by Biomedical Synergy LLC. Dr Amzad Hossain reports was provided by Biomedical Synergy LLC. Dr Amzad Hossain reports a relationship with Biomedical Synergy LLC that includes: employment. Dr Amzad Hssain has patent NA pending to NA. Dr Amzad Hossain If there are other authors, they declare that they have no known competing financial interests or personal relationships that could have appeared to influence the work reported in this paper.

## Data Availability

The data that has been used is confidential.

## References

[1] Francisco V., Figueirinha A., Costa G., Liberal J., Lopes M., García-Rodríguez C., Geraldes C., Cruz M., Batista M. (2014). Chemical characterization and anti-inflammatory activity of luteolin glycosides isolated from lemongrass. J. Funct. Foods.

[2] Tajidin N.E., Ahmad S.H., Rosenani A.B., Azimah H., Munirah M. (2012). Chemical composition and citral content in lemongrass (*Cymbopogon citratus*) essential oil at three maturity stages. Afr. J. Biotechnol..

[3] Basera P., Lavania M., Agnihotri A., Lal B. (2019). Analytical Investigation of *C. citratus* and Exploiting the Potential of Developed Silver Nanoparticle Against the Dominating Species of Pathogenic Bacteria. Front. Microbiol..

[4] Soltanzadeh M., Peighambardoust S., Ghanbarzadeh B., Mohammadi M., Lorenzo J. (2021). Chitosan nanoparticles encapsulating lemongrass (*Cymbopogon commutatus*) essential oil: Physicochemical, structural, antimicrobial and in-vitro release properties. Int. J. Biol. Macromol..

[5] Tak J., Jovel E., Isman M. (2015). Contact, fumigant, and cytotoxic activities of thyme and lemongrass essential oils against larvae and an ovarian cell line of the cabbage looper, Trichoplusia ni. J. Pest Sci..

[6] Manvitha K., Bidya B. (2014). Review on pharmacological activity of *Cymbopogon citratus.*. Int. J. Herb. Med..

[7] Shah G., Shri R., Panchal V., Sharma N., Singh B., Mann A.S. (2011). Scientific basis for the therapeutic use of *Cymbopogon citratus*, stapf (Lemon grass). J. Adv. Pharm. Technol. Res..

[8] 〈https://www.rxlist.com/lemongrass/supplements.htm〉. Visited on 28-06-2022.

[9] Sileshi S., Hassen A., Adem K. (2021). Drying kinetics of dried injera (dirkosh) using a mixed-mode solar dryer. Cogent Eng..

[10] Mishra D., Khare P., Singh D., Luqman S., Ajaya Kumar P., Yadav A., Das T., Saikia B. (2018). Retention of antibacterial and antioxidant properties of lemongrass oil loaded on cellulose nanofibre-poly ethylene glycol composite. Ind. Crops Prod..

[11] Francisco V., Figueirinha A., Costa G., Liberal J., Lopes M., García-Rodríguez C., Geraldes C., Cruz M., Batista M. (2014). Chemical characterization and anti-inflammatory activity of luteolin glycosides isolated from lemongrass. J. Funct. Foods.

[12] Balasubramanian D., Girigoswami A., Girigoswami K. (2022). Antimicrobial, pesticidal and food preservative applications of lemongrass oil nanoemulsion: a mini-review. Recent Adv. Food Nutr. Agric..

[13] Ali A., Ali A., Warsi M.H., Ahmad W., Amir M., Abdi S.A.H. (2023). Formulation of lemongrass oil (*Cymbopogon citratus*)-loaded solid lipid nanoparticles: an in vitro assessment study. 3 Biotech.

[14] Adhikary K., Banerjee P., Barman S., Bandyopadhyay B., Bagchi D. (2024). Nutritional Aspects, Chemistry Profile, Extraction Techniques of Lemongrass Essential Oil and It's Physiological Benefits. J. Am. Nutr. Assoc..

[15] Eid A.M., Naseef H., Jaradat N., Ghanim L., Moqadeh R., Yaseen M. (2023). Antibacterial and anti-acne activity of benzoyl peroxide nanoparticles incorporated in lemongrass oil nanoemulgel. Gels.

[16] Sahal G., Woerdenbag H.J., Hinrichs W.L.J., Visser A., Tepper P.G., Quax W.J., van der Mei H.C., Bilkay I.S. (2020). Antifungal and biofilm inhibitory effect of *C. citratus* (lemongrass) essential oil on biofilm forming by Candida tropicalis isolates; an in vitro study. J. Ethnopharmacol..

[17] De Silva B.C.J., Jung W.G., Hossain S., Wimalasena S.H.M.P., Pathirana H.N.K.S., Heo G.J. (2017). Antimicrobial property of lemongrass (*Cymbopogon citratus*) oil against pathogenic bacteria isolated from pet turtles. Lab Anim. Res.

[18] harma S., Habib S., Sahu D., Gupta J. (2021). Chemical properties and therapeutic potential of citral, a monoterpene isolated from lemongrass. Med. Chem..

[19] Brand-Williams (2013).

[20] Mclaughlin J.L., Rogers L.L., Anderson J.E. (1998). The use of biological assay to evaluate botanicals, Drug Info. Journal.

[21] Abdullah Al-Mqbali Latifa Rashid, Hossain Mohammad Amzad, Al Touby S. (2023). Study of cytotoxic and antibacterial potential of various varieties and polarities of extracts of unripe bananas. Carpathian J. Food Sci. Technol. (Scopus).

[22] Hossain Mohammad Amzad, Al Harthy Said, Said Salem, Al Touby Jaroof (2022). Review on phytochemicals and biological activities of natural sweeteners Stevia rebaudiana Bertoni. Int. J. Second. Metab..

[23] Al-Hajri Zahra Mohammed, Hossain Mohammad Amzad, Al Touby Salim Said (2022). Composition analysis and antibacterial activity evaluation of different crude extracts of Mentha piperita (Lamiaceae). Int. J. Second. Metab..

[24] Al-Abri Siham Saleh, Saida Sadri Abdullah, Said Al Touby Salem, Hossain Mohammed Amzad, Al-Sabahi Jamal Nasser (2022). Composition analysis and antimicrobial activity of essential oil from leaves of Laurus nobilis grown in Oman. J. Bioresour. Bioprod..

[25] Weli Afaf Mohammed, Ahmed Al-Abd Bayan Muhannad, Al-Saidi Anaam Humaid, Aljassasi Hajer Salim, Hossain Mohammad Amzad, Khan Ajmal, Numan Muhammad, Al-Jubouri Yasir (2022). Anil Phlip. The antibacterial, antioxidant and anti enzymatic activities of theleaves’ crude extracts of Hyoscyamus gallagheri. Adv. Biomark. Sci. Technol..

[26] (USEPA) U.S. Environmental Protection Agency. (1994). EPA Probit analysis program. Calculating LC/EC values Version 1.5. Retrived from (〈http://www.epa.gov/nerleerd/stat2.htm〉.

[27] Al-Dhahli Aaisha S., Al-Hassani Fatema A., Mohammed Alarjani Khaloud, Mohamed Yehia Hany, Al Lawati Wafa M., Najmul Hejaz Azmi Syed, Alam Khan Shah (2020). Essential oil from the rhizomes of the Saudi and Chinese *Zingiber officinale* cultivars: Comparison of chemical composition, antibacterial and molecular docking studies. J. King Saud. Univ. - Sci..

